# Phenotypic and molecular characterization of a set of tropical maize inbred lines from a public breeding program in Brazil

**DOI:** 10.1186/s12864-021-08127-7

**Published:** 2022-01-14

**Authors:** Sirlene Viana de Faria, Leandro Tonello Zuffo, Wemerson Mendonça Rezende, Diego Gonçalves Caixeta, Hélcio Duarte Pereira, Camila Ferreira Azevedo, Rodrigo Oliveira DeLima

**Affiliations:** 1 Department of Agronomy, Universidade Federal de Viçosa, Minas Gerais Viçosa, Brazil; 2 Department of Biology, Universidade Federal de Viçosa, Minas Gerais Viçosa, Brazil; 3 Department of Statistics, Universidade Federal de Viçosa, Minas Gerais Viçosa, Brazil

**Keywords:** Genetic relationships, Genetic diversity, Population structure, Linkage disequilibrium, Heterotic groups, Commercial hybrids

## Abstract

**Background:**

The characterization of genetic diversity and population differentiation for maize inbred lines from breeding programs is of great value in assisting breeders in maintaining and potentially increasing the rate of genetic gain. In our study, we characterized a set of 187 tropical maize inbred lines from the public breeding program of the Universidade Federal de Viçosa (UFV) in Brazil based on 18 agronomic traits and 3,083 single nucleotide polymorphisms (SNP) markers to evaluate whether this set of inbred lines represents a panel of tropical maize inbred lines for association mapping analysis and investigate the population structure and patterns of relationships among the inbred lines from UFV for better exploitation in our maize breeding program.

**Results:**

Our results showed that there was large phenotypic and genotypic variation in the set of tropical maize inbred lines from the UFV maize breeding program. We also found high genetic diversity (GD = 0.34) and low pairwise kinship coefficients among the maize inbred lines (only approximately 4.00 % of the pairwise relative kinship was above 0.50) in the set of inbred lines. The LD decay distance over all ten chromosomes in the entire set of maize lines with *r*^*2*^ = 0.1 was 276,237 kb. Concerning the population structure, our results from the model-based STRUCTURE and principal component analysis methods distinguished the inbred lines into three subpopulations, with high consistency maintained between both results. Additionally, the clustering analysis based on phenotypic and molecular data grouped the inbred lines into 14 and 22 genetic divergence clusters, respectively.

**Conclusions:**

Our results indicate that the set of tropical maize inbred lines from UFV maize breeding programs can comprise a panel of tropical maize inbred lines suitable for a genome-wide association study to dissect the variation of complex quantitative traits in maize, mainly in tropical environments. In addition, our results will be very useful for assisting us in the assignment of heterotic groups and the selection of the best parental combinations for new breeding crosses, mapping populations, mapping synthetic populations, guiding crosses that target highly heterotic and yielding hybrids, and predicting untested hybrids in the public breeding program UFV.

**Supplementary Information:**

The online version contains supplementary material available at 10.1186/s12864-021-08127-7.

## Background

Maize is one of the most important cereal crops grown for food, feed, and biofuel globally, and Brazil is the world’s third-largest maize producer after the United States and China [1]. Currently, hybrid maize accounts for over 95 % of the maize production area in Brazil and thus all Brazilian breeding programs of maize have focused their efforts in the development of hybrids cultivars. The basic fundamentals of a maize breeding program targeting hybrids, after the choice and improvement of germplasm (e.g. recurrent selection), are: (i) development of inbred lines by self-pollination and/or the double haploid method, (ii) selection of best inbred lines based on their *per se* and testcross performance, (iii) crossing between selected inbred lines from opposite heterotic groups, (iv) identification of hybrids that show consistent and reliable performance across several environments, and (v) commercial production of the best hybrids [2–4]. Consequently, understanding the genetic diversity and population differentiation of maize inbred lines from any breeding program will help breeders maintain and potentially increase genetic gain, resulting in high-yielding hybrids [5]. In this way, molecular markers have been extensively used to assess the genetic relationships, linkage disequilibrium (LD), population structure, and genetic diversity of breeding inbred lines in many maize breeding programs around the world. They have been successfully employed to: (i) assist in the selection of the best parental combinations for starting new pedigree populations [6–8], (ii) assign germplasm into heterotic groups [9–16], (iii) choose and replace testers [12, 17], (iv) predict untested hybrids and avoid crosses between similar inbred lines [18–23], and (v) estimate the loss of genetic diversity during the breeding process [24, 25]. According to Viana et al. [21], the overall LD in the parental lines has a significant impact on the prediction accuracy of non-assessed single cross performance, mainly for crops showing unclear heterotic groups, such as tropical maize.

The heterotic patterns in tropical maize are still unclear compared with the well-defined heterotic pattern Stiff Stalk x Non-Stiff Stalk present in temperate maize germplasm [6, 7, 11, 12, 26, 27]. The assignment of tropical maize inbred lines into heterotic groups began only three decades ago by the International Maize and Wheat Improvement Center (CIMMYT) based on the increasing maize hybrid adoption in tropical areas [7, 11, 12, 28]. Most of the tropical maize inbred lines were initially derived from broad germplasm pools, regardless of the racial origin or heterotic pattern [7, 12]. Moreover, the selfing of commercial hybrids, which is allowed in most countries, has been a very common practice to produce new inbred lines around the world, especially in Brazil [27, 29]. Consequently, tropical maize inbred lines have a complex and mixed genetic background, and their allocation into heterotic groups based on test cross performance with various representative testers has failed since the same line can be assigned to two or more clusters depending on the tester used, which might result in mixing up heterotic groups [5, 12, 27, 30]. In this scenario, population structure analysis using molecular markers have been used as an alternative approach to elucidate and allocate unknown and complex maize germplasms into heterotic groups [5, 12, 14, 28, 30–32]. According to Viana et al. [13], with an adequate number of molecular markers, population structure analysis is a very efficient method of assigning individuals to their populations of origin. Dinesh et al. [6] stratified a set of 64 tropical inbred lines from CIMMYT into three subpopulations by principal component analysis (PCA), and their results were consistent with the pedigree information. Segman et al. [7] assigned 450 tropical maize inbred lines from CIMMYT Africa into three clusters and found high concordance among the grouping based on the PCA and model-based population partition, and they suggested that the pattern of heterotic grouping based on both methods is more reliable than clustering analysis and phenotypic data. In another study, Ertiro et al. [11] allocated 265 tropical maize lines adapted to Ethiopia conditions into three clusters and concluded that the population structure inferred from molecular markers appeared to be more reliable than that from conventional methods based on the combination ability for grouping tropical maize germplasm.

In addition to population structure analysis, clustering analysis is also a very useful approach in maize breeding programs since an accurate dendrogram based on genetic distance and relationships among inbred lines can help breeders understand the genetic background of inbred lines, mainly among lines derived from commercial hybrids [5], and assist them in reducing the number of crosses to be made and evaluated [19, 31] and developing synthetic and breeding populations [33]. According to Larièpe et al. [34], the inclusion of the genetic distance between parental lines of hybrids in the model increased the general combining ability variance components for grain yield and other traits in maize. Thus, they suggested that this approach can be efficient for improving the estimates of the combining ability of inbred lines crossed with unrelated lines.

Association mapping based on LD provides a powerful tool for gene mapping in plants, and it has been widely used for genetic dissection of complex quantitative traits in several plant species since the first association study with flowering time and the *dwarf8* gene in maize was published [35]. Successful association mapping requires the creation of germplasm collection and a deep understanding of the genetic diversity, relatedness, population structure, and extent of LD decay of this germplasm [36–39]. An ideal association mapping panel needs to encompass suitable phenotypic variation and genetic diversity of the traits of interest and represents the breeding pool of the species for some growing conditions, such as the country, state, or target environment [37, 39]. According to Breseghello and Sorrels [40], the creation of an association panel from advanced breeding inbred lines with good field performance makes the process of introgression of identified genes or *QTLs (quantitative trait loci*) into breeding programs easier, faster, and more reliable than inbred line panels from exotic and poor field adaptation germplasms. Therefore, the balance between genetic diversity and germplasm adaptation must be considered in choosing maize inbred lines for the development of an association panel [41]. The extent of LD decay in the germplasm collection determines the resolution and power of the association mapping, and germplasms with rapid LD decay shohigher meiotic recombination rate and thus are more genetically diverse than high LD germplasms [37–39, 42]. Although elite breeding materials of many crops tend to have limited genetic diversity and slower LD decay due to domestication and strong breeding selection [43], LD decays rapidly in maize germplasm, and an association panel involving breeding inbred lines can provide adequate genetic diversity and resolution for QTL mapping, mainly for tropical maize [5, 35, 42, 44, 45]. The tropical maize germplasm has higher genetic diversity and shorter LD decay distance and contains more rare alleles than temperate maize [45–48]. However, although commercial hybrids are the main germplasm source for developing new lines in maize breeding programs in Brazil and other countries, previous studies have not performed genetic diversity and LD decay assessments among tropical inbred lines derived from the selfing of commercial hybrids.

Maize breeding in Brazil started in early 1930 at Universidade Federal de Viçosa (UFV), Viçosa (lat. 20°45’14"S; long. 42°52’55"W; alt. 648 m a.s.l.), Minas Gerais state. In 1938, the first commercial hybrid in Brazil was launched by UFV based on the research of Professors Gladstone Drummond and Antônio Secundino. In 1945, both researchers resigned from UFV and founded Agroceres, a private Brazilian seed company that was a leader in the commercial seed market for maize hybrids in Brazil for many years [49]. After half a century without important maize breeding activities, in the late 1990 s, the UFV maize breeding program was re-established, and today, it is the second largest and most important public maize breeding program in Brazil. After two decades of maize breeding, we constructed a germplasm collection of 187 inbred lines that represents the current UFV breeding pool. Most of those lines were derived from commercial hybrids planted in Brazil; therefore, they are composed of a mix of heterotic groups and have a complex genetic background. Currently, they have been characterized for nitrogen use efficiency and root morphology under greenhouse and field tropical conditions [50–52], although data are not available on the phenotypic and genetic diversity, heterotic patterns and LD decay degree present in these inbred lines. Thus, our objectives were to (i) evaluate whether this set of inbred lines comprises an association mapping panel for dissecting complex traits in tropical maize and (ii) investigate the population structure and patterns of relationships of maize inbred lines from UFV to better exploit their potential value in the development of hybrids, synthetic populations and new inbred lines as well as for developing heterotic groups.

## Materials and methods

### Plant material

A set of 187 inbred lines representative of tropical maize germplasm from the UFV breeding program was used in our study. This collection included 166 maize inbred lines derived from 49 commercial hybrids planted under tropical environments in Brazil, nine inbred lines derived from two open-pollinated varieties (BR106 and Nitroflint), and seven lines derived from two synthetic populations (CMS28 and CMS50; Table S1). It is important to highlight that in Brazil, it is permissible to use any commercial variety (hybrids or open-pollinated varieties) except those with transgenic events as a germplasm source for breeding purposes, such as for developing new inbred lines (10th article, third paragraph of the No. 9456 Brazilian Law of April 25, 1997). All sources of germplasm used in our program to develop new inbred lines were conventional hybrids, and all 187 inbred lines were developed using a modified pedigree method. Thus, our study complies with relevant institutional, national, and international guidelines and legislation.

### Phenotypic data

The phenotypic characterization of 187 inbred lines *per se* was carried out under field conditions across four environments: experimental station of the UFV, located in Coimbra, Minas Gerais State, Brazil, during the 2016 winter season (from January to June) and the 2016 to 2017 summer season (from October to April); and two experimental stations located in Viçosa, Minas Gerais State, Brazil, during the 2016 to 2017 summer season. Experiments were conducted under rain-fed conditions, and no irrigation was applied. Trial management was the same for all experiments and employed standard agricultural practices. In all environments, the trial was laid out in an alpha-lattice incomplete design with two replications. Each plot was a single 4-m row, with rows spaced 0.80 m apart. Plots were overseeded with hand planters and then thinned at the V3 stage to a plant population of 62,500 plants ha^-1^.

We evaluated a physiological plant trait (chlorophyll concentration), a set of plant architecture traits and yield components, and grain yield. Ear leaf chlorophyll concentration (SPAD) was measured 15 d after silking in the middle of the ear leaf using a SPAD-502 chlorophyll metre (Minolta Camera Co., Osaka, Japan). For plant architecture, ten traits were measured: days to pollen (DTP), days to silking (DTS), ear leaf length (LL, cm), ear leaf width (LW, cm), ear leaf area (LA, cm^2^), plant height (PH, cm), ear height (EH, cm), stalk diameter (SD, mm), and above and below ear node number (AENN and BENN, respectively). For the yield components, six traits were measured: number of ears per plant (EPP), ear length (EL, cm), number of kernel rows (NKR), ear (ED, cm) and cob diameter (CD, mm), and one thousand-kernel weight (TKW, g) as the weight of 1,000 kernels adjusted to 145 g kg^-1^ moisture. Finally, grain yield (GY) was recorded for all ears in the plots at physiological maturity. The ears were shelled, the grain weight and grain moisture percentage were recorded, and GY (kg ha^-1^) was calculated at 145 g kg^-1^ moisture.

### Genotypic data

For the molecular characterization, we genotyped 182 inbred lines out of 187 evaluated for phenotypic traits. Leaf tissue samples were obtained from the bulk of five plants for each inbred line and sent to DuPont Pioneer® Company, where DNA extraction and genotyping were performed. All 182 inbred lines were genotyped using the GoldenGate platform (Illumina, San Diego, CA, USA), containing 3,713 SNPs distributed across 10 chromosomes [53]. The SNP information used in this study was found in the studies by Jones et al. [54], Gore et al. [55], and Ganal et al. [56]. Of the 3,713 SNPs genotyped, a set of 3,083 SNPs with a minor allele frequency (MAF) greater than 0.01 and a 90 % call rate was selected for genetic characterization analysis of all inbred lines in this study.

## Phenotypic data analyses

A mixed model implemented in the R package “lme4” [57] was used to estimate the variance components and predict the genotypic values of each inbred line across environments. Environment and replication were included in the model as fixed effects, whereas block and inbred line were considered random effects. The interactions between fixed and random effects were included in the model as random effects. The phenotypic values were modelled according to the following equation: ${ \text{y}}_{\text{i}\text{r}\text{m}\text{k}}={\upmu }+{{\upalpha }}_{\text{i}}+{{\upgamma }}_{\text{k}}+{{\upalpha }{\upgamma }}_{\text{i}\text{k}}+{{\upphi }}_{\text{r}\left(\text{k}\right)}+{{\updelta }}_{\text{m}\left(\text{r}\text{k}\right)}+{{\upepsilon }}_{\text{i}\text{k}\text{r}\text{m}}$, where ${\upmu }$ is the overall mean, ${{\upalpha }}_{\text{i}}$ is the random effect of the inbred line, ${{\upgamma }}_{\text{k}}$ is the fixed effect of the environment, ${{\upalpha }{\upgamma }}_{\text{i}\text{k}}$ is the random effect of the inbred line-by-environment interaction, ${{\upphi }}_{\text{r}\left(\text{k}\right)}$ is the fixed effect of replication within the environment, ${{\updelta }}_{\text{m}\left(\text{r}\text{k}\right)}$ is the random effect of the block within replication within the environment, and ${{\upepsilon }}_{\text{i}\text{k}\text{r}\text{m}}$ is the random effect of error.

Variance components were estimated by using a restricted estimation of maximum likelihood, and genotypic values of inbred lines across environments were predicted using the best linear unbiased predictors (BLUPs) [58]. A likelihood ratio test deviance analysis was used to test random effects via the chi-square statistic [59]. Ranges and mean values were based on BLUPs. Broad-sense heritability (ĥ^2^) on an inbred line-mean basis was estimated for each trait as follows [2]: ${\widehat{\text{h}}}_{\stackrel{-}{\text{x}}}^{2}=\frac{{\widehat{{\upsigma}}}_{\text{G}}^{2}}{{\widehat{{\upsigma}}}_{\text{G}}^{2} +^{{\widehat{{\upsigma }}}_{\text{G}\text{x}\text{E}}^{2}}\!/\!{\text{n}} +^{{\widehat{{\upsigma }}}^{2}}\!/ \!{\text{n}\text{r}}}$, where ${\widehat{{\upsigma }}}_{\text{G}}^{2}$, ${\widehat{{\upsigma }}}_{\text{G}\text{x}\text{Y}}^{2}$, and ${\widehat{{\upsigma }}}^{2}$ are the genotypic variance estimates, variance estimates due to the inbred line-by-year interaction, and error variance estimates, respectively; and n and r are the number of environments and replications, respectively. Pearson’s correlation coefficients between pairs of traits were estimated based on the BLUP of each trait using the R “agricolae” package.

The genetic diversity assessment among the inbred lines based on phenotypic data was performed using the R “ade4” and “adegenet” packages. We generated the distance matrix based on the Mahalanobis generalized distance, which accounted for residual correlations among traits [60]. Then, a Mahalanobis distance matrix was used as input data for a clustering analysis based on the unweighted pair-group method of arithmetic average (UPGMA). The UPGMA dendrogram was generated based on the Mahalanobis generalized distance to estimate the level of relatedness among inbred lines using the R “ape” package [61]. The Mojena [62] method was used to allocate the inbred lines into clusters. According to this method, the dendrogram must be cut as a function of the mean value of the genetic distance of fusion levels and the standard deviation of the distance values.

## Genotypic data analysis

We used the filtered dataset of 3,083 SNPs to estimate the allele frequency, polymorphic information content (PIC), gene diversity, heterozygosity rate and pairwise relative kinship among inbred lines. The allele frequency analysis of the 3,083 SNPs was carried out using TASSEL 5.0 software [63]. The PIC values of these 3,083 SNPs were calculated in Microsoft Excel 2016 according to the following formula: ${\widehat{PIC}}_{l}=1-\sum _{u=1}^{k}{\tilde{P}}_{lu}^{2}-\sum _{u=1}^{k-1}{\sum }_{v=u+1}^{k}2{\tilde{P}}_{lu}^{2}{\tilde{P}}_{lv}^{2}$, where ${\tilde{P}}_{lu}^{2}$ and ${\tilde{P}}_{lv}^{2}$ are the frequencies of the *u*th and *v*th alleles of marker *l*, respectively, and the summation extends over *k* alleles [64]. The gene diversity (GD) and heterozygosity rate for the 3,083 selected SNPs were estimated using the R “Poppr” package [65]. GD was defined as the probability that two alleles randomly chosen from a population are different. It was estimated at each locus as $\widehat{\text{D}}=^{(1-\sum _{u=1}^{k}{\tilde{P}}_{lu}^{2})}\!/ \!_{(1+\frac{1+f}{n})}$, where ${\tilde{P}}_{lu}^{2}$ is the frequency of the *u*th allele, *n* is the sample size, and *f* is the inbreeding coefficient [66]. Finally, the pairwise relative kinship among the 182 inbred lines was estimated with the 3,083 selected SNPs using the “Centred IBS” (identity by state) method proposed by Endelman and Jannink [67] and implemented in TASSEL 5.0 [63] software.

In addition to genetic diversity based on phenotypic data (analysis detailed above), we also assessed the genetic diversity from molecular data. Nei’s genetic distance [68] between inbred lines was assessed with the 3,083 selected SNPs using the R “adegenet” package [69]. Then, the UPGMA dendrogram for all 182 maize inbred lines was generated based on their Nei’s genetic distances using the R “ape” package [69]. The Mojena [62] method was also used to allocate the inbred lines into clusters.

The population structure of the 182 inbred lines was investigated using STRUCTURE software [70] and principal component analysis (PCA) based on 3,083 SNPs. In the first approach, an admixture model-based clustering method implemented in Structure was run for K clusters, ranging from 1 to 10, and each K was run 20 times, with a burn-in period of 10,000 and 100,000 Markov Chain Monte Carlo (MCMC) replications. The ad hoc statistic delta K (ΔK) was used to determine the most likely number of clusters [71] using Structure Harvester software [72]. The software Clumpak [73] was used to align grouping labels across the twenty runs before plotting the data. Allele frequencies were assumed to be correlated, and loci were assumed to be unlinked. Inbred lines with membership probabilities higher than or equal to 0.60 were assigned to the same cluster, while those with membership probabilities lower than 0.60 were assigned to a “mixed” cluster [7, 12, 41, 47]. In the second approach to infer the structure population, a PCA based on 3,083 SNPs was performed using the R “pcaMethods” package [74], and the first two principal components were illustrated for the visual examination of the clustering pattern of inbred lines.

The LD between each pair of SNPs on each chromosome was estimated by the square Pearson correlation coefficient (*r*^2^), with 3,083 SNPs using TASSEL 5.0 software [63]. The average LD decay distance within and over ten chromosomes with *r*^*2*^ = 0.1 was used to measure the difference in LD decay distance between all inbred lines. A 50-kb slide window was used to determine the width of the window on one side of the start site, the spacing between two loci on the same chromosome was segmented at a distance of 50 kb, and the average LD was assessed for each window. The LD values between each pair of SNPs were plotted using the R “ggplot2,” “Mass,” and “scales” packages [75–77].

## Results

### Phenotypic variation and genetic parameters based on phenotypic traits

Considerable variation was observed for all measured traits in this set of maize inbred lines, as shown by the wide ranges of genotypic values across four environments (Table 1). Days to flowering (DTP and DTS) ranged from approximately 66 to 73 days. Plant height and EH ranged from 102.1 to 170.2 cm and 40.5 to 100.6 cm, respectively. Leaf length, LW, and LA ranged from 60.2 to 96.6 cm, 7.3 to 12.7 cm, and 401.7 to 710.3 cm^2^, respectively. Concerning the yield components, NKR and TKW ranged from 9.7 to 21.9 rows and from 158.7 to 350.1 g, respectively, and grain yield ranged from 1,208.8 to 3,605.9 kg ha^− 1^. The variance components associated with the inbred lines were highly significant (*P < 0.01*) based on the likelihood ratio test for all traits. The variance components due to inbred line × environment interactions were significant (*P < 0.05*) for seven of 18 measured traits, namely, LW, PH, EH, EL, ED, TKW, and GY; therefore, the inbred lines had different relative performances across environments for these traits. In general, the estimates of broad-sense heritability (${\widehat{\text{h}}}_{\stackrel{-}{\text{X}}}^{2}$) were intermediate to high and ranged from 0.44 (EPP) to 0.79 (DTS).

**Fig. 1:**
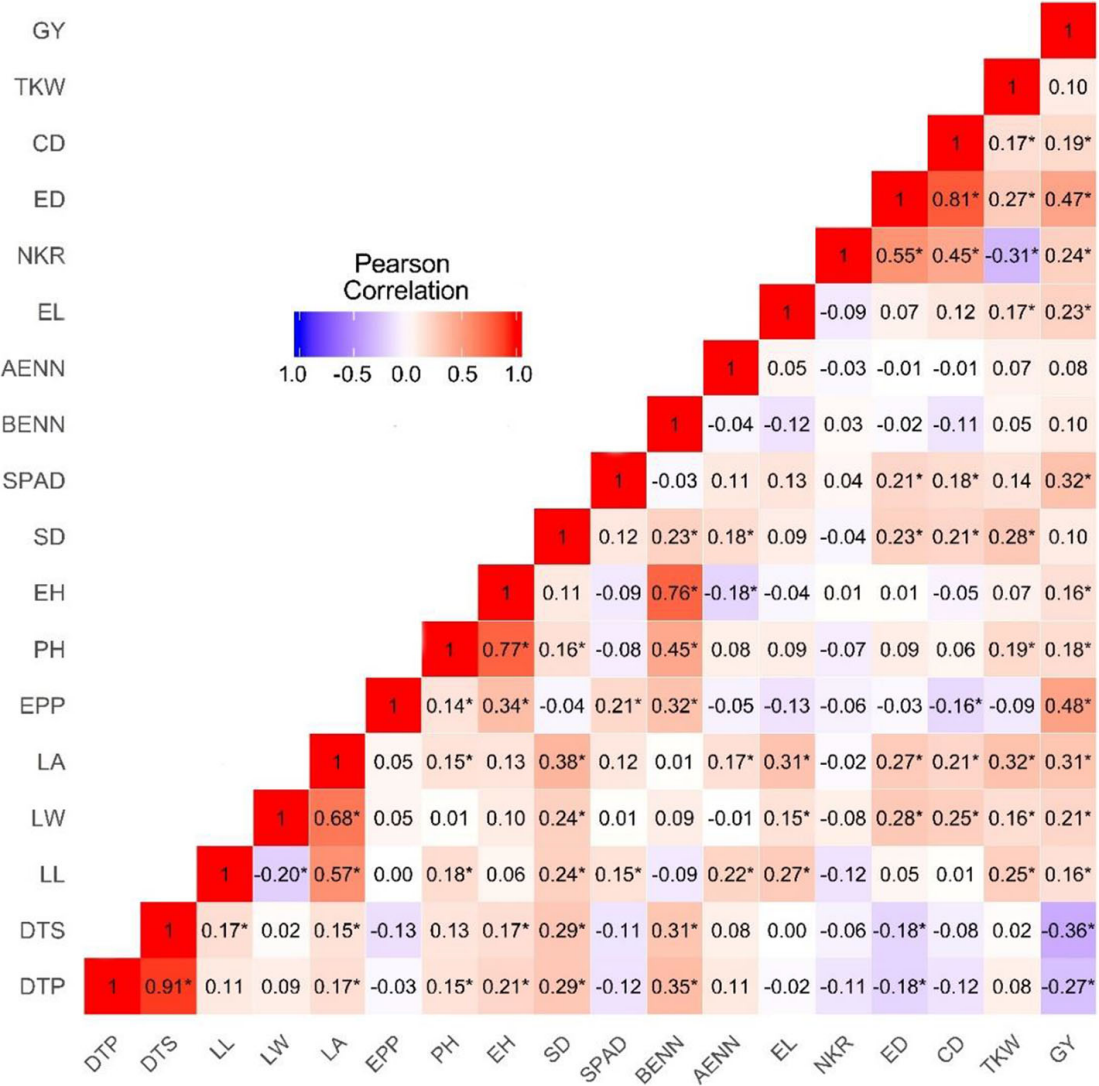
Heat map of Pearson correlation coefficients between pairs of vectors of genotypic values of the traits measured in this set of tropical maize lines across four environments. The color assigned to a point in the Heat map grid indicates the strength of a correlation between two traits. The level of correlation is indicated by red for positive correlations and blue for negative correlations, as depicted in the color key. * Significant at *P* = 0.05

Although most Pearson correlation coefficients among traits were not significant (*P > 0.05*) or showed low magnitude, we found moderate-to-strong positive (*r* > 0.45) correlations for some pairs of traits (Fig. 1). Strong correlations were observed between DTP and DTS (*r* = 0.91), CD and ED (*r* = 0.81), EH and PH (*r* = 0.77), and EH and BENN (*r* = 0.76), whereas moderate correlations were found between BENN and PH (0.45), between LA and LL (0.57) and LW (0.68), between NKR and CD (0.45) and ED (0.55), and between GY with ED (0.47) and EPP (0.48). Grain yield showed low or no correlation with the other tested traits.

**Table 1:** Best linear unbiased prediction estimates of ranges and means, estimates of variance components due to inbred lines (${\widehat{{\upsigma }}}_{\text{G}}^{2}$) and inbred lines x environments interaction (${\widehat{{\upsigma }}}_{\text{G}\text{E}}^{2}$), broad-sense heritability estimates (${\widehat{\text{h}}}_{\stackrel{-}{\text{X}}}^{2}$) and coefficient of variation (CV%) for 18 traits measured in this set of tropical maize lines across four environments

Traits^1^	Min.	Mean	Max.	${\widehat{{\upsigma }}}_{\text{G}}^{2}$	${\widehat{{\upsigma }}}_{\text{G}\text{E}}^{2}$	${\widehat{\text{h}}}_{\stackrel{-}{\text{X}}}^{2}$	CV%
DTP	66.2	72.8	80.8	9.1***^2/^	0.5^ns^	0.79	2.5
DTS	65.8	73.0	80.1	9.3***	0.6^ns^	0.76	2.7
SPAD	30.3	44.1	55.0	25.8***	1.5^ns^	0.64	11.9
LL	60.2	76.3	96.6	39.1***	6.8^ns^	0.58	9.2
LW	7.3	9.3	12.7	0.8***	0.1*	0.67	8.7
LA	401.7	529.9	710.3	3229.0***	712.2^ns^	0.53	13.4
PH	102.1	136.8	170.2	249.3***	21.1*	0.75	8.8
EH	40.5	71.7	100.6	133.5***	13.7***	0.76	11.9
SD	17.4	21.3	26.1	3.4***	0.3^ns^	0.59	9.8
BENN	5.5	7.1	9.4	0.5***	0.1^ns^	0.66	9.8
AENN	4.4	5.9	7.4	0.3***	0.0^ns^	0.68	8.6
EPP	0.8	1.1	1.4	0.0***	0.0^ns^	0.44	18.3
EL	10.1	13.1	16.9	1.8***	0.3*	0.67	8.0
NKR	9.7	13.9	21.9	3.4***	0.2^ns^	0.76	8.8
ED	30.5	37.5	45.2	7.5***	1.8***	0.69	5.1
CD	16.0	23.4	28.8	5.3***	0.3^ns^	0.76	6.4
TKW	158.7	239.0	350.1	1314.6***	335.0***	0.71	9.8
GY	1028.8	2263.3	3605.9	289,626***	94,102***	0.47	28.6

^1/^*DTP* days to pollen (days), *DTS* days to silking (days), *SPAD* ear leaf chlorophyll concentration, *LL* leaf length (cm), *LW* leaf width (cm), *LA* leaf area (cm^2^), *PH* plant height (m), *EH* ear height (m), *SD* stalk diameter (mm), *BENN* bellow ear node number, *AENN* above ear node number, *EPP* number of ears per plant, *EL* ear length (cm), *NKR* number of kernel rows, *ED* ear diameter (mm), *CD* cob diameter (mm), *TKW* one thousand kernel weight (g), and grain yield (GY, g kg^− 1^)

^2/^*** Significant at *P* = 0.01, ** significant at *P* = 0.05, * significant at *P* = 0.10 and ^ns^ not significant by the likelihood ratio test

### Summary statistics of SNP markers

After filtering the total SNP data, 3,083 high-quality SNPs with a minor allele frequency (MAF) greater than 0.01 and a 90 % call rate were selected for further genetic characterization analysis. The number of SNPs showed moderate variation among the 10 maize chromosomes and ranged from 207 on chromosome 10 to 494 on chromosome 1, with an average of 308 SNPs per chromosome (Table 2). The heterozygosity rate ranged from 1.16 % on chromosome 3 to 2.61 % on chromosome 2, with an average of 1.97 %, and the PIC ranged from 0.26 on chromosomes 1 and 6 to 0.35 on chromosome 8, with an average of 0.28. MAF and DG showed little variation among chromosomes, with averages of 0.25 % and 0.34, respectively.

**Table 2:** The chromosomal distribution markers (SNPs), missing rate (%), minor allelic frequency (MAF), gene diversity, heterozygosity rate (%), and polymorphic information content (PIC) per chromosome in the entire set of 182 tropical maize inbred lines

Chromosome	SNPs per Chr.	Missing rate (%)	MAF (%)	Gene Diversity	Heterozygosity rate (%)	PIC
1	494	0.93	0.24	0.32	2.10	0.26
2	340	1.03	0.25	0.34	2.61	0.27
3	369	0.84	0.25	0.33	1.61	0.27
4	355	0.98	0.26	0.35	1.82	0.28
5	304	0.87	0.25	0.33	1.16	0.27
6	247	0.87	0.24	0.32	2.31	0.26
7	254	0.84	0.25	0.34	2.15	0.35
8	277	0.96	0.27	0.35	2.19	0.28
9	236	1.00	0.24	0.33	1.62	0.27
10	207	1.04	0.26	0.34	2.15	0.27
Average	308.30	0.94	0.25	0.34	1.97	0.28

### Relative kinship

**Fig. 2:**
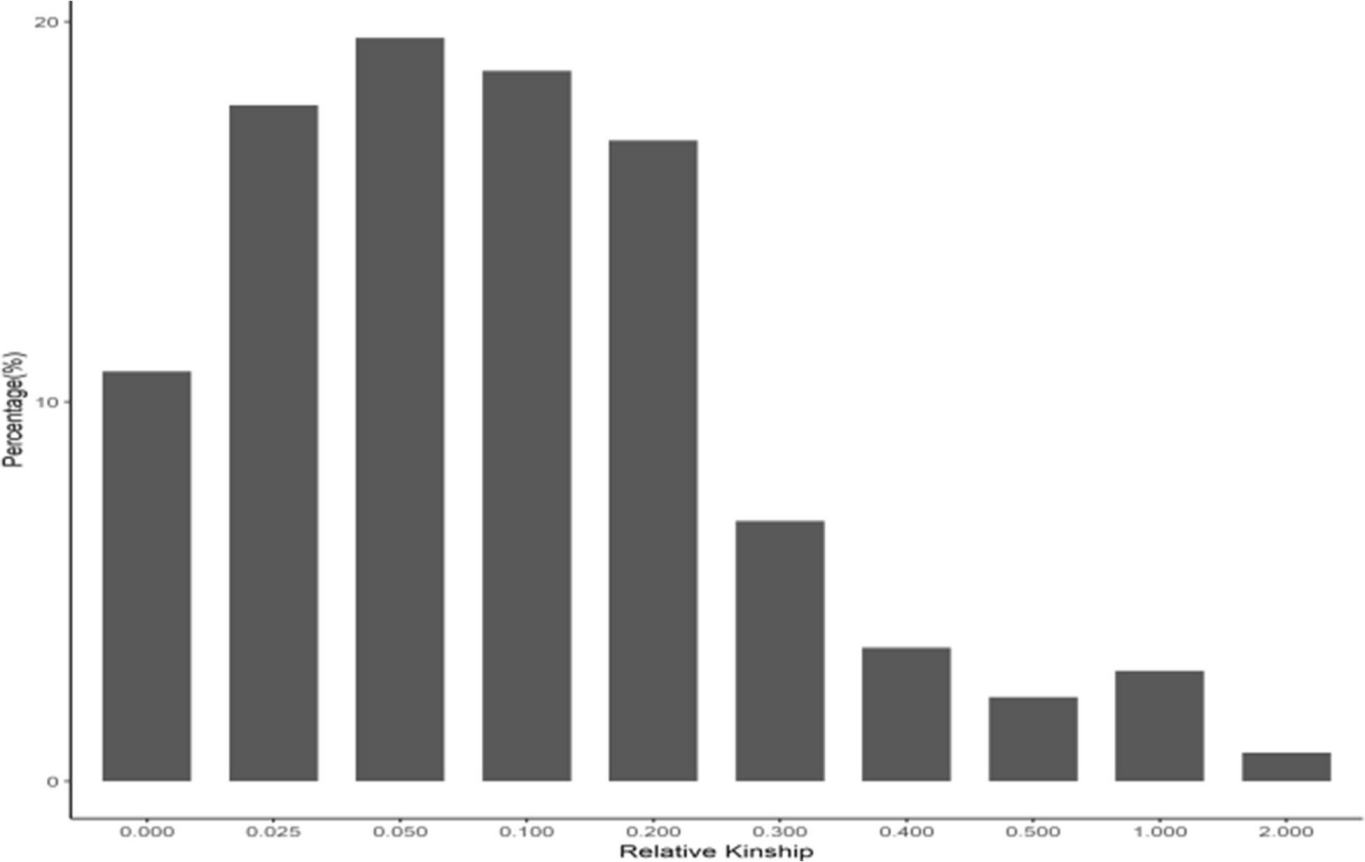
Distribution of pairwise relative kinship for 182 tropical maize inbred lines calculated using 3,083 filtered SNP markers

The pairwise relative kinship coefficients among the 182 tropical maize inbred lines ranged from 0.00 to 2.00, but their distribution between 0.00 and 0.50 represented 96.34 % of the data (Fig. 2). The results showed that 10.80 % of the pairwise relative kinship was equal to 0; 66.88 % ranged from 0.00 to 0.10; 48.16 % ranged from 0.10 to 0.50; and only approximately 4.00 % were above 0.50. This pattern of genetic relatedness indicated that most inbred lines were weakly or moderately related to each other, with only a few lines showing strong similarities among them.

### LD decay

**Fig. 3:**
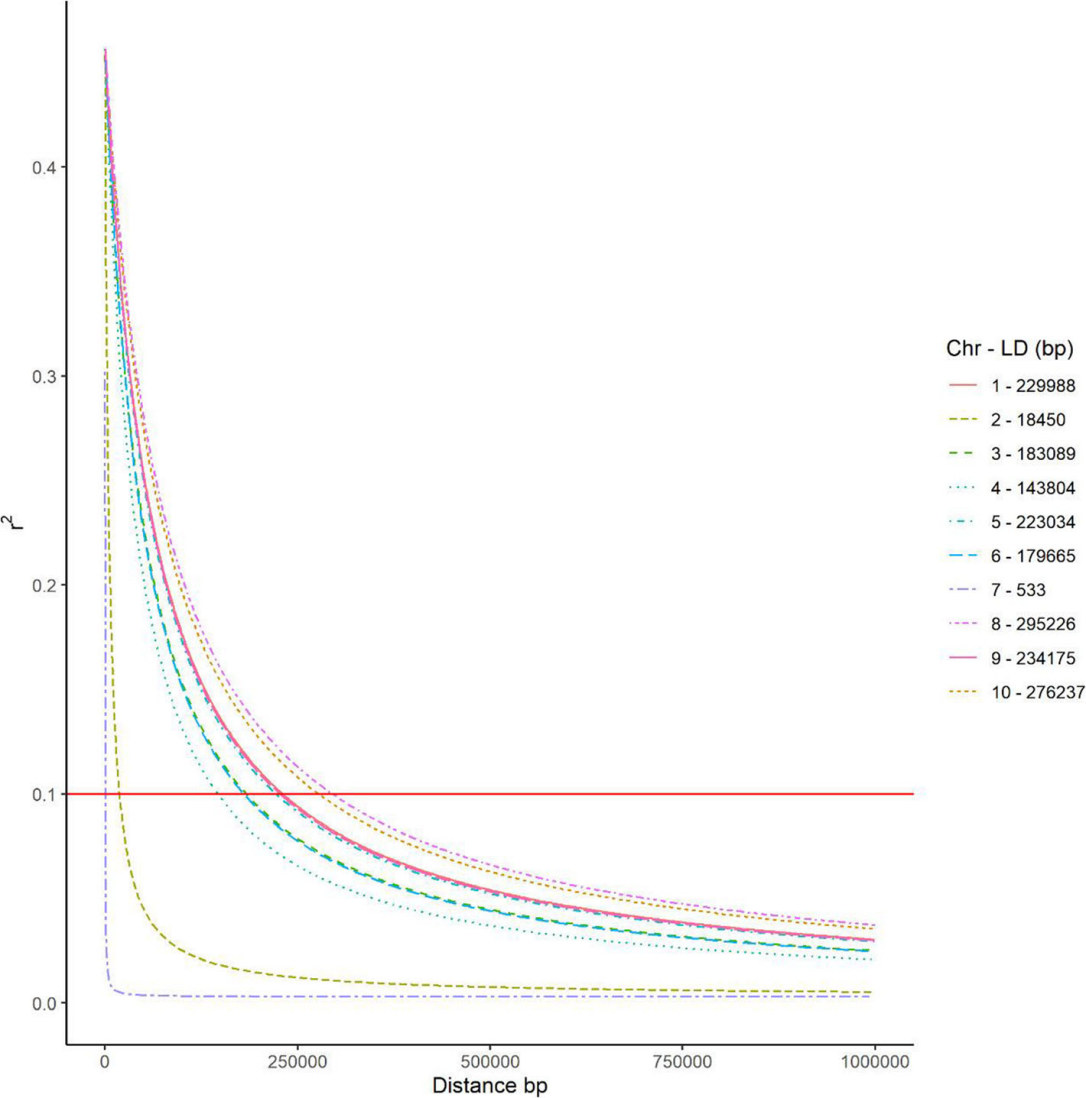
Whole-genome linkage disequilibrium in the entire 182 tropical maize inbred lines. Linkage disequilibrium within and over chromosomes is given in physical distance of 10 kb

The average LD decay distance over all ten chromosomes in the entire set of inbred lines with *r*^*2*^ = 0.1 was 276,237 kb, and it ranged from 533 bp (on chromosome 7) to 295,226 bp (on chromosome 8; Fig. 3). The average LD decay distance was very similar across eight chromosomes, but a relatively fast LD decay distance was observed on chromosomes 7 (533 bp) and 2 (18,450 bp).

### Population structure

**Fig. 4:**
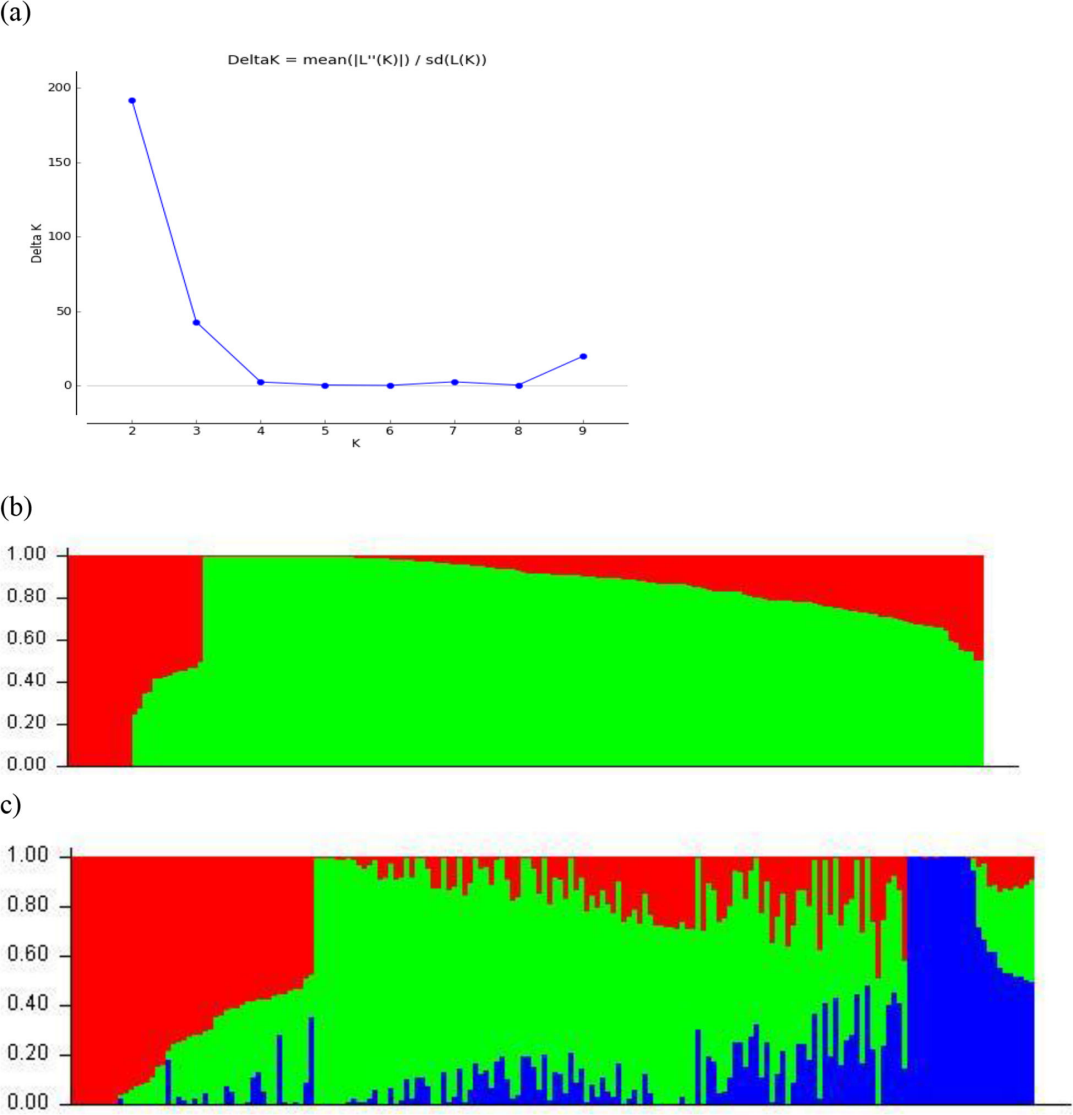
Analysis of the population structure of 182 tropical maize inbred lines using SNPs markers. **a** Δk (delta K) values for K ranging from 1 to 10. **b** Estimated population structure of 182 tropical maize inbred lines as revealed by 3,083 SNP markers for k = 2, and **c** for k = 3 assessed by STRUCTURE. In STRUCTURE, each maize inbred line is represented by a thin vertical bar, which is partitioned into three colored segments on the x-axis with the lengths proportional to the estimated probability membership on the y-axis. For all classes, a given subpopulation is represented: *Green*, subpopulation 1; *Red*, subpopulation 2; *Blue*, subpopulation 3

According to the results of our population structure analysis based on the STRUCTURE model, the most significant peak of Δ*k* was observed when *k* = 2, with a sharp decrease when *k* increased from 2 to 4 (Fig. 4a). However, at *k* = 3, Δ*k* was significantly higher than at *k* = 4; therefore, *k* = 2 and *k* = 3 can be considered the best possible numbers of subpopulations, and the set of lines could be divided into two or three subpopulations. The first level of clustering (*k* = 2) separated the lines into subpopulations 1 and 2, with 141 (77.5 %) and 25 (13.7 %) maize inbred lines allocated into each subpopulation, respectively, and a mixed subpopulation (membership probabilities < 0.60) with 16 (8.8 %) inbred lines (Fig. 4b). When *k* = 3, subpopulations 1, 2, and 3 consisted of 84 (46.2 %), 47 (25.8 %), and 21 (11.5 %) inbred lines, respectively, and the remaining 30 (16.5 %) inbred lines were classified into a mixed subpopulation (Fig. 4c). When the maize inbred lines were allocated into three subpopulations, subpopulations 1, 2, and 3 consisted of inbred lines derived from 35, 24, and 13 commercial hybrids, respectively. Although some maize hybrids contributed inbred lines to more than one subpopulation, in general, inbred lines derived from hybrids of the same seed company tended to be clustered in the same subpopulation. Concerning the broad-based source populations, all seven inbred lines derived from Nitroflint, three out of four from CMS50, and two out of four from CMS28 were clustered into subpopulation 1, whereas the other CMS50 and CMS28 lines were clustered into subpopulation 2. The two inbred lines derived from BR106 were clustered into a mixed subpopulation.

**Fig. 5:**
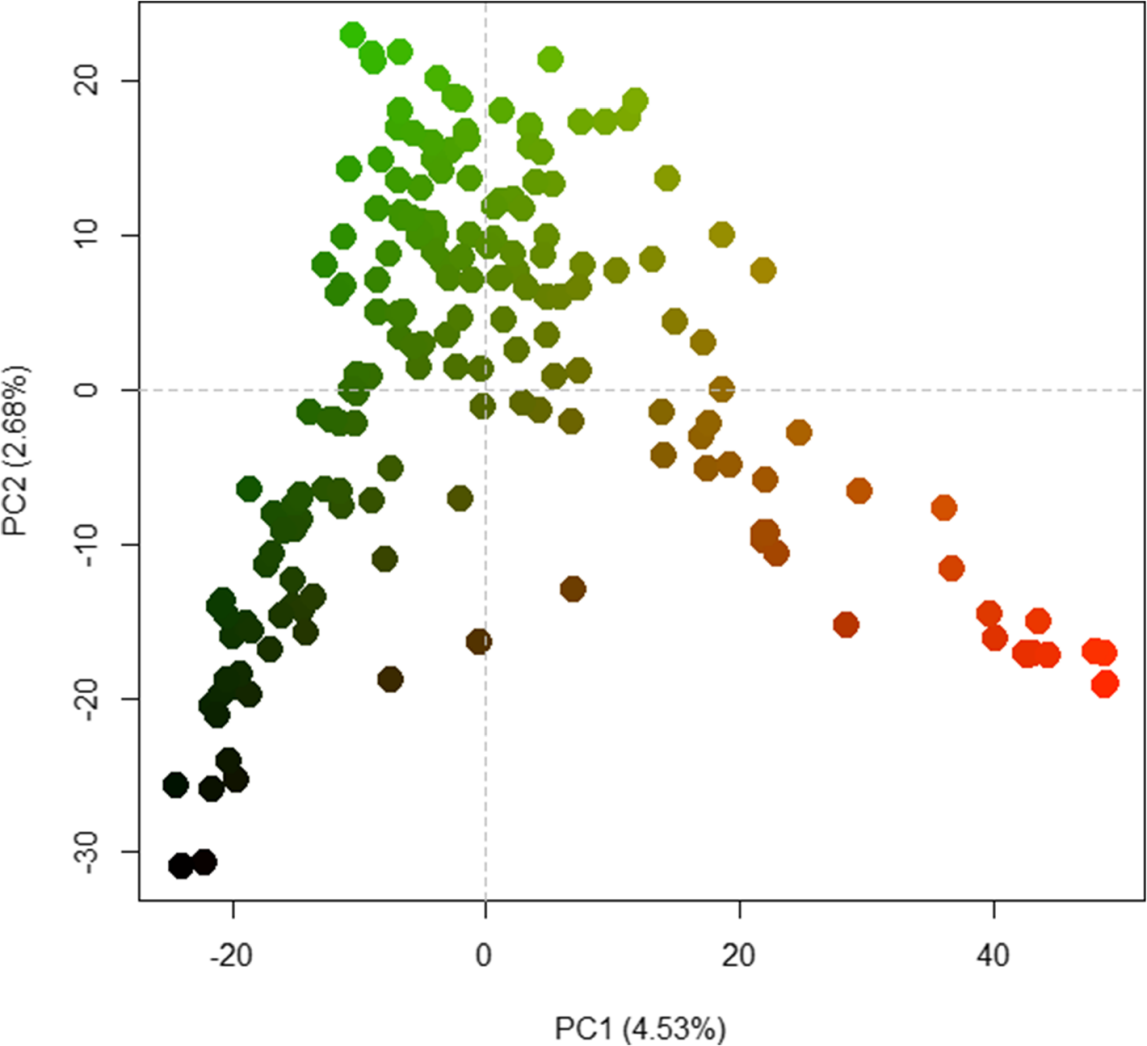
Plot of PC1 (4.53 %) and PC2 (2.68 %) from principal components analysis (PCA) estimated with 3,083 SNP markers of 182 tropical maize inbred lines

Regarding the population structure based on the PCA, the first two principal components (PCs) explained 7.21 % of the total SNP variation in the entire set of lines and the 182 inbred lines were clearly distinguished into three subpopulations (Fig. 5). Grouping based on the PCA was very consistent with the STRUCTURE results, and subpopulations 1 (98 lines), 2 (54 lines), and 3 (30 lines) consisted of all maize inbred lines from subpopulations 1, 2, and 3 from the STRUCTURE results, respectively (Table S4). Furthermore, the 30 inbred lines considered mixed by STRUCTURE were separated into three subpopulations based on PCA, and thus fourteen, seven, and nine lines from the mixed subpopulation were allocated into subpopulations 1, 2, and 3, respectively.

### Genetic diversity

**Fig. 6:**
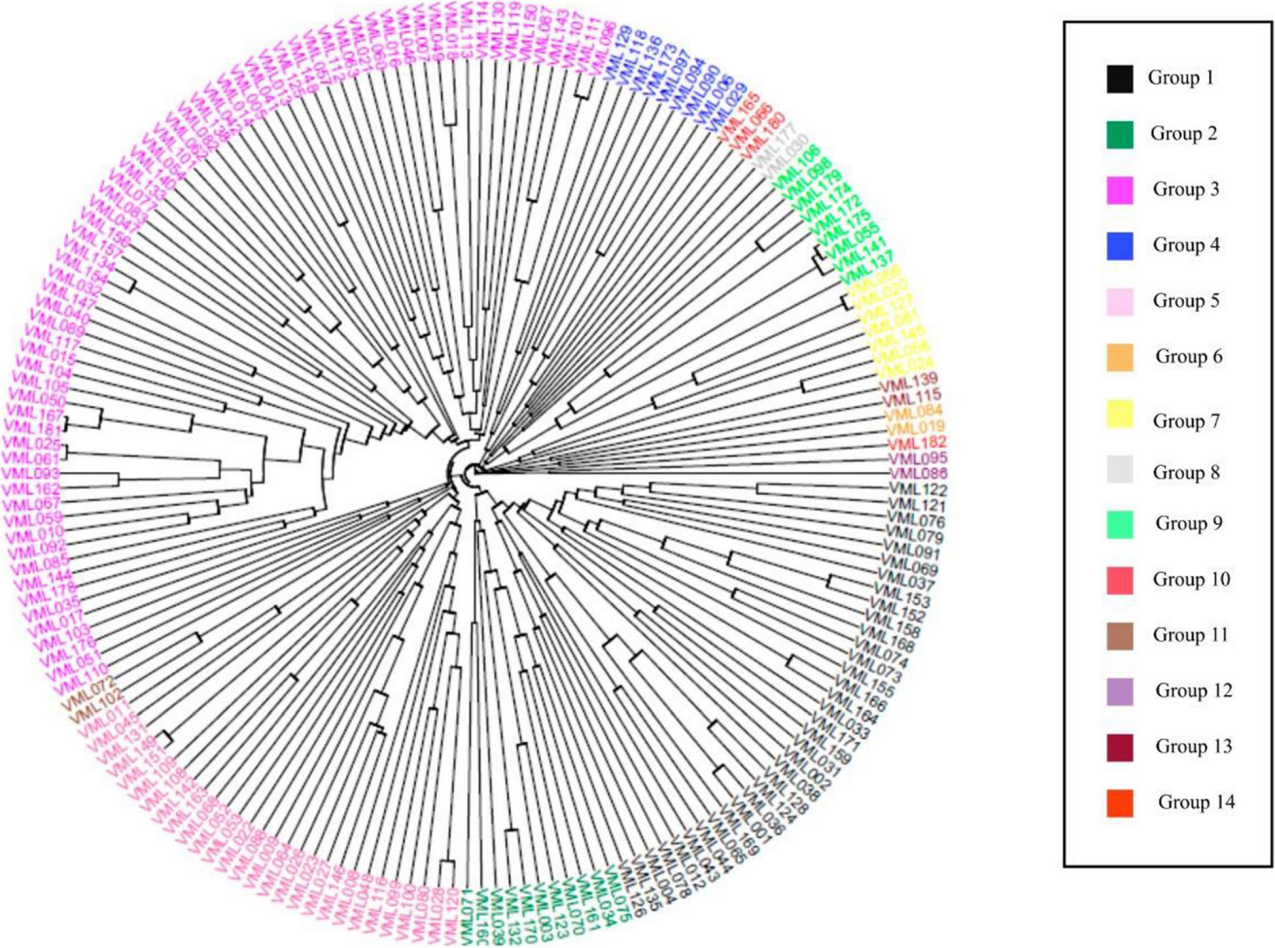
UPGMA Dendrogram using genotypic data. Dendrogram from unweighted pair-group method of arithmetic clustering for 182 maize inbred lines using Nei’s genetic distances calculated from 3,083 SNP markers

The mean Nei’s genetic distance among lines was 0.400, and it ranged from 0.003 (between VML001 and VML036) to 0.480 (VML006 and VML079), and the UPGMA dendrogram grouped the 182 inbred lines into 14 clusters based on Mojena’s method ([62]; Fig. 6). VML006 and VML079, which were derived from hybrids AG4051 and BALU184, respectively, were the most distant lines, and they were allocated into clusters 1 and 2, respectively, whereas the most similar lines (VML001 and VML036) were allocated into cluster 1. Interestingly, similar inbred lines were derived from different maize hybrids (30F87 and AG1051, respectively). The genetic diversity clusters 3, 1, and 5, the largest clusters, consisted of 69, 35, and 28 inbred lines, respectively. Clusters 6 (VML019 and VML084), 8 (VML030 and VML177), 11 (VML072 and VML102), 12 (VML086 and VML095), 13 (VML115 and VML139), with two inbred lines each, and 14 (VML182), with one line, were the smallest genetic diversity clusters. Nearly all these clusters consisted of inbred lines derived from different commercial hybrids except VML030 and VML177, which were derived from hybrid P3041. Overall, the inbred lines derived from the same hybrid showed a slight trend to be grouped in the same cluster. Moreover, all inbred lines derived from hybrids BRS1010 (2 lines), DKB199 (3), C333 (7), and Z8420 (4) were grouped together into clusters 3, 1, 3, and 3, respectively, and nearly all lines derived from Balu184 (6 out of 9 lines), AG8080 (5 out of 6), Balu551 (4 out 7), DKB435 (5 out of 6), and P30F90 (6 out of 7) and from population Nitroflint (5 out of 7) were grouped together in the same clusters. In contrast, the 16 inbred lines derived from hybrid P3041, seven inbred lines derived from P30F87, and six inbred lines derived from Garra were grouped into seven, six, and four different clusters, respectively. We also found moderate concordance between the results of molecular marker clustering and population structure in terms of group members. All lines clustered into genetic diversity cluster 1 were allocated into subpopulation 2 based on the STRUCTURE and PCA results, and nearly all inbred lines from clusters 4, 5, and 7 as well as all lines from clusters 6, 10, 11, 12, 13, and 14 were allocated into subpopulation 1. Moreover, all inbred lines from genetic diversity cluster 3 were allocated into subpopulation 3.

**Fig. 7:**
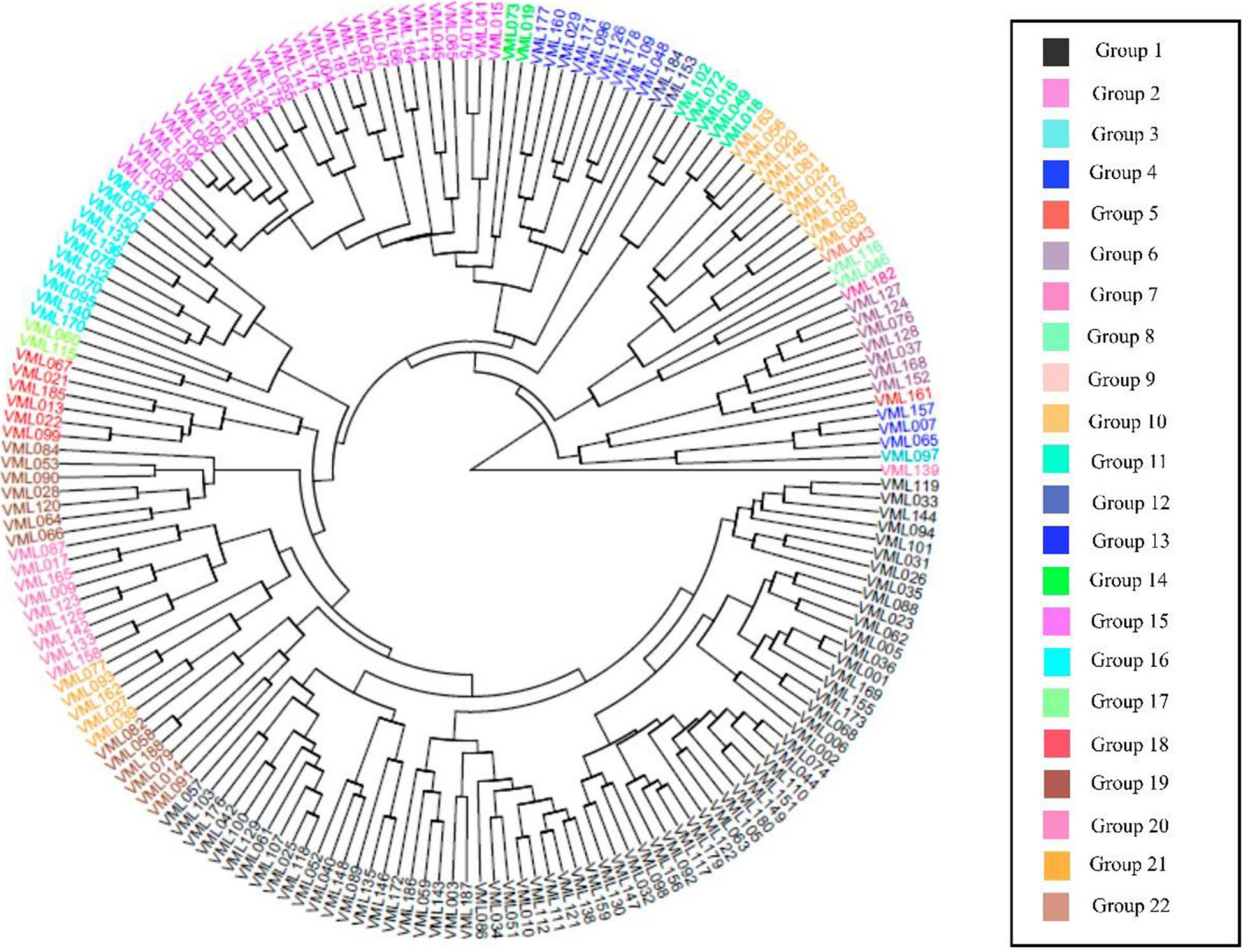
UPGMA Dendrogram using phenotypic data. Dendrogram from unweighted pair-group method of arithmetic clustering for 187 tropical maize inbred lines using Mahalanobis distance based on 18 traits across four environments

In relation to phenotypic diversity, the mean Mahalanobis distance was 61.81, and the values ranged from 4.66 (VML001 and VML036) to 219.46 (VML089 and VML1170); in addition, the clustering analysis placed the 187 maize inbred lines into 22 clusters (Fig. 7). The inbred lines VML089 and VML170, the most distant lines, were allocated into clusters 1 and 2, respectively, whereas VML001 and VML036, the most similar inbred lines, were allocated into cluster 1. These inbred lines also presented the shortest distance and were allocated in the same cluster based on the molecular clustering analysis. Clusters 1 and 2 were the largest clusters and consisted of 68 and 28 inbred lines, respectively, whereas clusters 14 (VML043), 18 (VML097), 19 (VML139), 21 (VML161), and 22 (VML182) consisted of only one inbred line each. VML182 also grouped alone into a cluster based on molecular marker clustering. Interestingly, approximately 50 % (35 lines) of inbred lines grouped into cluster 1, and all lines from clusters 7 (5 lines) and 11 (7 lines) were allocated into subpopulation 1 based on the population structure results. Although almost 50 % (32 lines) of inbred lines grouped into cluster 1 based on phenotypic data were allocated in the same cluster (3) based on the molecular data, there was low concordance between the clustering of inbred lines based on both data sets. Furthermore, we did not observe correspondence between the germplasm source and the clustering of inbred lines based on phenotypic data, which was observed for molecular marker clustering.

## Discussion

Comprehensive phenotypic and molecular characterization of the breeding inbred lines for genetic diversity, population structure, genetic relationships, LD decay distance and field adaptation is a prerequisite for maize breeders to define the best breeding strategies to increase the genetic gain in a breeding program and evaluate whether a set of breeding lines can be used in an association-mapping panel. In our study, we found substantial genotypic variation for all tested traits, and most inbred lines had good field performance for agronomic traits across tropical environments. The intermediate to high ${\widehat{\text{h}}}_{\stackrel{-}{\text{x}}}^{2}$ values found for the traits, even for those traits that presented significant inbred line × environment interactions, indicated that accurate estimates of genetic effects can be obtained in future QTL experiments with this set of lines. The huge phenotypic variation presented for agronomic traits in our set of lines and the ${\widehat{\text{h}}}_{\stackrel{-}{\text{x}}}^{2}$ values observed for some traits were similar to those reported in other maize association panels [39, 41, 78, 79]. Furthermore, some traits appear to be genetically controlled by the same genomic regions (pleiotropic effects) or genes that are at LD (closely linked or not) since some pairs of traits showed moderate-to-strong correlations. Thus, those associated traits should be genetically dissected based on the trait that showed the greater ${\widehat{\text{h}}}_{\stackrel{-}{\text{x}}}^{2}$ value.

Although the average GD (0.34) observed in our set of inbred lines was lower than the GD value of 0.39 found by Yang et al. [41] across 527 global diverse lines that were representative of tropical, subtropical and temperate germplasm, our average GD was similar to the GD values (approximately 0.35) found across two sets of elite inbred lines used in Chinese breeding programs [46, 47] and a set of diverse CIMMY inbred lines from Africa [7]; and much higher than the GD values (average GD ranging from 0.22 to 0.32) reported at five sets of inbred lines consisting of temperate and tropical maize [12, 14, 28, 79, 80], across 94 tropical inbred lines (GD = 0.27) used in the breeding program of Brazilian Agriculture Research Corporation (Embrapa) [28], and across 94 tropical maize inbred lines adapted to West and Central Africa (GD = 0.22) [10]. Moreover, our inbred lines presented larger genetic diversity than the P group (GD = 0.21), a Chinese heterotic group that consists of inbred lines derived from modern US hybrids [47]. Thus, abundant genetic diversity was observed in our set of lines, which was primarily due to the broad genetic base present in the commercial hybrids used as a germplasm source for developing most of those lines. According to Andrade [25], large genetic variability occurs in the maize germplasm used by commercial breeding programs in Brazil.

The pairwise kinship coefficients among the 182 inbred lines of UFV collection were low and clearly indicate the lack of redundant breeding inbred lines in the UFV collection. When we compared our results with kinship coefficients reported in other breeding inbred lines adapted to specific environments, they were similar to those observed among 367 breeding lines used in Chinese breeding programs [47], 226 inbred lines derived from the Suwan population and temperate resources [81], and 265 tropical inbred lines developed by three public companies and adapted to Ethiopia conditions [11]. Moreover, our coefficients were lower than those observed among 450 inbred lines from the CIMMYT breeding programs in Africa [7] and among 157 advanced breeding lines from the breeding program in Sichuan Province, China [30]. Conversely, they were higher than pairwise kinship coefficients reported in two global maize collections, which included 538 inbred lines representing a tropical maize collection developed by CIMMYT [12] and a set containing 281 temperate and 351 tropical and subtropical lines [45]. In these studies, the authors reported that approximately 60 % of pairwise kinship coefficients were equal to zero, whereas only 10.80 % were equal to zero. However, they evaluated the global maize collection of highly diverse inbred lines that were derived from broad-based populations and adapted to different environments, which contributed to the low level of relatedness among inbred lines in their studies. In contrast, our inbred lines represented only the UFV breeding pool, most of them were derived from commercial hybrids, and beyond our lines are adapted to specific Brazilian tropical conditions.

The LD decay distance is influenced by the source of inbred lines and selection intensity during the breeding process; hence, the values tend to be considerably higher in the breeding germplasm than in more diverse germplasm collections [5, 36, 38, 42, 44, 82]. Consistent with this finding, the average LD decay distance (276,237 bp) in our set of 182 lines was much larger than those observed in diverse maize collections around the world, where several studies reported LD decay ranging from 5 kb in most collections containing temperate and tropical germplasm to 100 kb in a set of 283 ex-PVP inbred lines from North American elite commercial maize germplasm [5, 6, 12, 45, 48, 81]. The slower LD decay in our set of inbred lines can be attributed to their complex genetic background due to recent mixture of heterotic groups since the inbred lines were derived from commercial hybrids and tropical populations with mixed origin, and experienced limited opportunities for recombination. However, the LD decay in our lines is consistent with results reported in other breeding germplasms, including 391 kb in a set of 367 inbred lines widely used in maize breeding in China [47], approximately 1,000 kb (range of 500 to 1,500 kb) in two sets (157 and 362) of inbred lines from the current Southwest China breeding programs [30, 46], and in a set of 1,537 elite inbred lines from the plant breeding company Limagrain [44]. Thus, the breeding inbred lines of UFV programs are more diverse and contain more rare alleles than elite inbred lines from most maize breeding programs around the world.

As the inbred lines of the UFV program were derived from commercial hybrids and populations with mixed origin, they are constituted from a mixture of tropical heterotic groups. Furthermore, some of them also contain temperate alleles in their genetic constitution due to the introgression of temperate germplasm in Brazil that has been performed by private seeds companies over the last 25 years. Consequently, our inbred lines had a very complex genetic background that cannot be attributed to major tropical heterotic groups, e.g., Tuxpeño and non-Tuxpeño (ETO, Cateto, Caribbean, Suwan, Tuson, etc.) recently recognized by CIMMYT [27, 28, 83]. However, heterotic groups can be created and enhanced by breeders through reciprocal pedigree recurrent selection schemes for combining ability among groups of inbred lines defined by the population structure analysis using molecular markers [10, 13, 15–17, 31]. In our study, the population structure results revealed that the inbred lines of UFV may be placed into two or three well-defined groups. Thus, due to the high concordance among the PCA and model-based population partition in terms of members of each group and the assignment of tropical maize inbred lines into three or more putative heterotic groups proposed in other studies using population structure analysis [6, 7, 11, 12, 19], we considered three major groups for future exploitation of these inbred lines in our hybrid breeding program. Moreover, Adu et al. [10] recently assigned 94 inbred lines derived from populations with mixed origins into three clusters and concluded that SNP markers clearly allocated the inbred lines into three heterotic groups.

According to our results, our breeding proposal with inbred lines of UFV is to explore the heterosis among identified clusters and develop well-established heterotic groups from these three clusters. We intend to explore heterosis among the three groups by crossing a set of inbred lines with desirable agronomic traits from different groups to determine the combining ability among inbred lines and to develop hybrids with high heterotic and yielding hybrids for Brazilian tropical conditions. Here, we recommend crosses between grouped inbred lines that are very divergent from inbred lines from opposite groups based on SNP genetic distances, since hybrid performance is higher when parents are genetically diverse [19, 23, 84–86]. Then, heterotic hybrids must be evaluated across several tropical environments in Brazil, and information on combining ability may be used to complement the grouping of inbred lines based on molecular markers, choice of testers and in the development of breeding populations and new inbred lines. To increase genetic divergence among groups and develop heterotic groups, we proposed the implementation of reciprocal recurrent selection (RRS) schemes among synthetic populations from different clusters [87–91]. Thus, maize synthetic populations from each group will be used as base populations to begin an RRS among groups and, consequently, derive new inbred lines using the synthetic populations and their improved version as a germplasm source. However, many generations of RRS may be necessary to increase the heterotic response among groups and develop well-established heterotic groups among the three major groups of lines present in the UFV collection. In addition to synthetic populations, the less divergent inbred lines with good field performance within each group may be crossed in pairs to produce biparental populations for the development of inbred lines using pedigree breeding and/or the double haploid method. According to Hallauer et al. (2010), the use of F2 genetically narrow-based populations made from elite x elite-related lines is the most common procedure for maize inbred line development. Finally, the inbred lines developed from biparental and/or synthetic populations from a group should be crossed and tested with one or more testers from the opposite group.

In relation to clustering analysis, the variable levels of genetic distance between pairs of inbred lines associated with several genetic divergence clusters based on both molecular (14) and phenotypic (22) data suggest the presence of a low percentage of redundant inbred lines that contribute very little to the genotypic variation and diversity in the UFV collection, thus indicating that our inbred lines are largely diverse. The low correlation between the clustering based on phenotypic and molecular data observed in our study is consistent with the results reported in previous studies on maize [92, 93], and it should not be considered a limitation to assess genetic diversity among breeding accessions in a germplasm bank. In fact, the assessment of genetic diversity based on phenotypic data provides additional information that is generally independent of genotypic information and represents a complementary tool that must be combined with molecular data to assist in breeding program efforts [29, 31, 89]. According to Silva et al. [19], SNP-based genetic distances provide important insights for selecting the best parental combinations within and among heterotic groups in tropical maize, whereas Hansey et al. [79] recommended that a maize panel intended for association mapping must utilize the maximum phenotypic diversity possible.

## Conclusions

We found large genotypic variation and abundant genetic diversity in the set of tropical maize inbred lines from UFV breeding program, and the results indicated that these lines can be exploited for breeding purposes. Our results also showed that there are low kinship coefficients and high genetic distance (based on phenotypic and genotypic data) among most pairs of inbred lines as well as short LD decay distance in the entire set of breeding lines, which clearly indicates the uniqueness of most inbred lines. Thus, our maize inbred lines can be used to construct an association panel for genome-wide association studies performed to dissect the variation in complex quantitative traits in maize, mainly in tropical environments. Concerning applied breeding, we identified three major groups based on the population structure analysis, and along with the results of the molecular marker clustering analysis, these findings will be useful for exploring the heterosis among lines, guiding crosses, selecting testers, developing highly heterotic and yielding hybrids for Brazilian tropical environments as well as for establishing heterotic groups in the UFV breeding program.

## Supplementary Information

**Additional file 1: Table S1. **Germplasm summary.

**Additional file 2: Table S2.** SNP Summary.

**Additional file 3: TableS3.** Predicted genotypic means for agronomic traits of 182 tropical maize inbred lines evaluated across four environments. Brazil.

**Additional file 4: TableS4.** Population structure and genetic diversity summary of 187 tropical maize inbred lines.

## Data Availability

The datasets generated and/or analyzed during the current study are available in the Figshare repository, 10.6084/m9.figshare.13625648.v1.
